# Characterization of COVID-19’s Impact on Mobility and Short-Term Prediction of Public Transport Demand in a Mid-Size City in Spain

**DOI:** 10.3390/s21196574

**Published:** 2021-09-30

**Authors:** Ana Belén Rodríguez González, Mark R. Wilby, Juan José Vinagre Díaz, Rubén Fernández Pozo

**Affiliations:** Group Biometry, Biosignals, Security, and Smart Mobility, Departamento de Matemática Aplicada a las Tecnologías de la Información y las Comunicaciones, Escuela Técnica Superior de Ingenieros de Telecomunicación, Universidad Politécnica de Madrid, Avenida Complutense 30, 28040 Madrid, Spain; abrodriguez@etsit.upm.es (A.B.R.G.); mrwilby@etsit.upm.es (M.R.W.); juanjose.vinagre@upm.es (J.J.V.D.)

**Keywords:** Bluetooth traffic monitoring system, COVID-19, prediction, public transport, smart card data, smart mobility

## Abstract

COVID-19 has dramatically struck each section of our society: health, economy, employment, and mobility. This work presents a data-driven characterization of the impact of COVID-19 pandemic on public and private mobility in a mid-size city in Spain (Fuenlabrada). Our analysis used real data collected from the public transport smart card system and a Bluetooth traffic monitoring network, from February to September 2020, thus covering relevant phases of the pandemic. Our results show that, at the peak of the pandemic, public and private mobility dramatically decreased to 95% and 86% of their pre-COVID-19 values, after which the latter experienced a faster recovery. In addition, our analysis of daily patterns evidenced a clear change in the behavior of users towards mobility during the different phases of the pandemic. Based on these findings, we developed short-term predictors of future public transport demand to provide operators and mobility managers with accurate information to optimize their service and avoid crowded areas. Our prediction model achieved a high performance for pre- and post-state-of-alarm phases. Consequently, this work contributes to enlarging the knowledge about the impact of pandemic on mobility, providing a deep analysis about how it affected each transport mode in a mid-size city.

## 1. Introduction

In December 2019, the world started facing an unprecedented global crisis caused by the new coronavirus (COVID-19). This virus spread so fast that the World Health Organization officially declared the COVID-19 as global pandemic only three months later, in March 2020. One year later, COVID-19 has infected more than 115 million people globally and caused more than 2.56 million deaths [1], with Spain being one of the most strongly affected European countries. COVID-19 has become a major global issue with large socio-economic and health impacts. Because of the severity of the pandemic, governments throughout the world limited or stopped every non-essential economic and commercial activity. These decisions resulted in important changes in people’s usual life and behavior: remote work was promoted, unnecessary journeys were restricted, educational institutions migrated to online teaching strategies, and events and public gatherings were canceled. These drastic changes led mobility to collapse worldwide. Previous scientific studies have performed macroscopic analyses of this massive downfall. These overall studies often use partial information collected from phone data or surveys. Whilst serving the purpose of their macroscopic focus, they do not provide detailed information on specific transport modes (the former) and are based on small samples that could be potentially biased (the latter). Detailed analyses would require data-driven approaches, but the corresponding scientific literature is still very limited. These exhaustive results would also provide a solid background to support prediction models, which could eventually contribute to optimize public transport services in order to avoid overcrowded areas and vehicles and the subsequent risk of infection.

In the light of these considerations, the aim of this paper is two-fold. First, we perform an extensive data-driven analysis of the impact that the preventive lockdown measures in Spain had on mobility, particularized for every private and public transport mode in a representative mid-size city (Fuenlabrada, Spain) in Madrid’s metropolitan area. Second, we develop a short-term predictor of public transport demand adapted to the sequence of phases within the course of the pandemic: severe restrictions in the outbreak, deescalation, and “new normality”. To this effect, we used real public transport and private vehicle data collected from the Smart Card Data (SCD) system operating in the region and a Bluetooth Traffic Monitoring System (BTMS) deployed as a living-lab in Fuenlabrada, respectively. Thus, our findings contribute to fill the knowledge gap regarding the pandemic’s impact on weekly and daily mobility patterns in mid-size cities, specific to each transport mode, and propose short-term predictors of future public transport demand.

The remainder of the paper is structured as follows. Section 2 reports the related work about how pandemic has affected mobility worldwide, highlighting the previous works focused on analyses within specific cities. Section 3 presents the case study we used in our work, describing the city’s main features, the phases in the pandemic, and the data we used. Section 4 specifies the methodology we followed for the study of the impact of COVID-19 on mobility, displays the obtained results, and discusses the findings we reached. Section 5 states how short-term traffic demand predictors were constructed and their corresponding results. Finally, Section 6 is devoted to providing the fundamental conclusions and future research of our work.

## 2. Related Work

At the time of writing this paper, the COVID-19 pandemic is still active. The early research on its impact on different fields of life can be categorized into four major themes, (1) environmental quality, (2) socio-economic factors, (3) management and governance, and (4) transportation and urban design [2]. The first two are clearly dominant, whilst the latter is yet to be explored in depth. In this respect, we observe that only a very limited number of detailed studies about the consequences of the pandemic on internal mobility within cities have been found. Among this reduced set, the work [3] reported an integrated analysis of the mobility changes that the pandemic produced in Santander, Spain. Data were collected from traffic counters, public transport, intelligent transportation systems (ITS), and traffic control cameras to establish comparisons between flows and travel times before and during the confinement. The results revealed an overall 76% decline in mobility, which reached 93% in the case of public transport users. In addition, the authors in [4] presented a data-driven analysis of the changes in mobility volume and modal distribution in Budapest, Hungary, due to COVID-19. This work also revealed a steeper drop in public transport, which was substituted by road transport and other individual and environmentally friendly modes such as cycling. The authors raised an important question regarding the temporal or long-term nature of the observed changes. Apart from these two interesting references, the impact of COVID-19 on mobility has been mainly investigated from a global perspective, using data aggregated at national or regional level.

This set of works adopt two main approaches depending on the data sources they use. A first group uses mobile phone tracking such as Google Mobility Report [5], or mobile application management including Apple Mobility Trends [6] or Moovit App [7]. These location data were employed to examine the effect of COVID-19 and safer-at-home policies on mobility patterns and trends during, before, and after lockdown periods in different regions and countries throughout the world: India [8], Colombia [9] Portugal [10], Italy [11], USA [12], and Japan [13]. At a city level, [14] interpreted the change in Sapporo’s population density during the emergency period, using big data obtained from mobile spatial statistics. Results indicated that the city’s residents were more likely to stay home and less likely to travel downtown. The effects of mobility habits in the spread of COVID-19 and vice versa have been studied in other interesting previous works. In [15], the incidence of citizen mobility regarding the spread of the pandemic was investigated, showing that the number of daily certified cases of COVID-19 infections is strongly linked with trips made 21 days before. This finding was confirmed in [16], where the authors analyzed the mobility behavior using data from cellular networks through a specific use case in Málaga (Spain), showing a correlation between mobility and number of cases. Using data from Apple mobility trends, the relation between human mobility tendencies and severe COVID-19 outcomes was also studied in [17], concluding that the reduction in mobility had a significant impact on decreasing COVID-19 mortality, thus providing crucial evidence in support of such government’s preventive measures. Other interesting reference can be found in [18], where the authors analyzed the mobility behavior during the six months of lockdown in Metro Manila (Philippines), using aggregated cell phone and GPS (Global Positioning System) data from Google and Apple. However, all these studies show an important drawback given that the location data they use do not represent actual public transport and private road trips nor include specific and precise information about transport modes.

A second group of works base their studies on mobility surveys conducted worldwide. As a common finding, all of them show an important reduction in public and private mobility. For example, the ongoing series of surveys designed to identify the changing patterns in travel activity of Australian residents as a result of the restrictions can be found in [19,20]. In [21], insights about the effects of the virus and the Dutch government’s “intelligent lockdown” on people’s activities and travel behavior were presented using data from survey panels. Their main findings show that approximately 80% of the population reduced their outdoor activities, with a deeper decrease observed in elderly people and those inclined to use private cars instead of public transport. Other interesting works following this approach can be found in [22,23,24,25]. Nevertheless surveys may not be an appropriate approach to generate a comprehensive knowledge about travelers’ behavior in a city as they just include a small percentage of the whole population.

With respect to data-driven studies, we can find specific works on private vehicle and public transport that treat them separately, thus lacking of an integrated view. The former focuses on analyzing the impact of the pandemic and the subsequent restrictions on traffic flows as in [26] (Rome, Italy), [27] (Qatar), [28] (several locations in the UK), and [29] (Slovakia), and its positive effects on pollution [26,30,31], and accidents [32]. Among the works that study the pandemic’s impact particularly related with public transport using data-driven analyses, let us highlight the following research. The study in [33] analyzes the impacts of COVID-19 on daily public transport ridership in the three most populated regions of Sweden. In [34], the effect of the “dynamic lockdown” strategy adopted in Chile on public transport demand was studied. Authors in [35] study the changes in the city bus network of A Coruña, Spain (ridership, use of stops, operation time, etc.) during the COVID-19 lockdown and the “new normality” phases. Finally, the impact of the pandemic on micro-mobility services was analyzed in [36], showing that trips remarkably decreased during the lockdown period in Zurich.

Apart from the evident contribution of this set of works in understanding the changes observed in mobility due to the pandemic and the subsequent restrictions, no prediction model was capitalized from this massive knowledge base. Potential prediction models could clearly benefit the public transport operation and management avoiding overcrowded situations in indoor spaces, which imply an ideal scenario for the expansion of the virus. Over the last years prior to the outbreak of the pandemic, numerous studies focusing on public transport ridership prediction have emerged, supported by the availability of SCD in cities worldwide [37]. These short-term transportation forecasting approaches can be generally divided into two categories: parametric and non-parametric techniques [38]. Among the traditional parametric techniques, historical average, smoothing techniques and Autoregressive Integrated Moving Average (ARIMA) have been the most popular approaches to predict transportation demand. On the other hand, neural networks have been frequently adopted as the modeling approach for non-parametric techniques due to their adaptability to different conditions [38]. Other remarkable references included in this group are: in [39], the prediction was performed using a multiscale radial basis function (MSRBF) network, [40] employed gradient boosting decision trees, and [37] analyzed forecasts of the public transport ridership to infer passenger behavior.

The great majority of the preceding literature analyzed COVID’s impact on mobility in big cities. However, we observe a limited number of studies reporting a detailed and integrated analysis about mobility changes in mid-size cities during the COVID-19 pandemic. In addition, our work uses specific data from both private and public transport and builds specific models for each phase of the pandemic. Consequently, our research contributes to extend the knowledge about COVID’s impact on mid-size cities, providing a better understanding of local mobility patterns during the pandemic outbreak in one of the hardest hit countries. Furthermore, prior to the outbreak of the pandemic, none of the works proposing prediction models for future public transportation ridership had to face a key factor such as COVID-19, which fundamentally impacted the usual pattern behaviors. In this respect, our investigation extends its first analytic results and constructs particularized predictors of future mobility demands in each phase of the pandemic, which entails a second significant contribution to the scientific literature in this area. According to this, we believe that the conclusions and results of this paper will be highly valuable to operators and transit agencies who will gain insights of the effects of critical situations on transportation together with forecasting tools that predict future demands, which would eventually support them optimizing their services.

## 3. Case Study Description

Fuenlabrada is a city located in the Community of Madrid, Spain. As of 2018, it has a population of 193,586, making it the region’s fourth most populated municipality. The city has experienced a great growth related to the economic development of Madrid, centered on industry and the service sector. Fuenlabrada represents 25% of the whole regional industrial business, which results in an important mobility activity from and to this city. Despite this fact, Fuenlabrada has an average income of 22,691 Euro per capita and year (2017), one of the lowest within the region.

**Figure 1:**
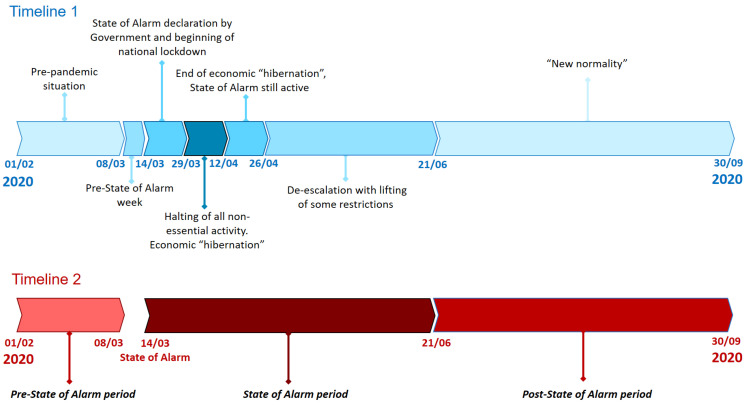
Different phases of COVID-19 timeline in Spain.

The pandemic hit Spain on 31 January 2021 and rapidly developed throughout the country. As a preventive response to the evolution of the pandemic, the Spanish government imposed a sequence of restrictions that directly impacted mobility, which we show in Timeline 1 in Figure 1. These phases can be grouped in 3 distinct periods according to the state of alarm declared in the country, as it is shown in Timeline 2 in Figure 1, which we will employ to extract average daily patterns and perform predictions.

In order to implement our study, we collected two different datasets of public transport and private vehicle data, extending from 10 February 2020 to 30 September 2020 (8 months), which we describe in the following subsections.

### 3.1. Public Transport Network and Dataset

**Figure 2:**
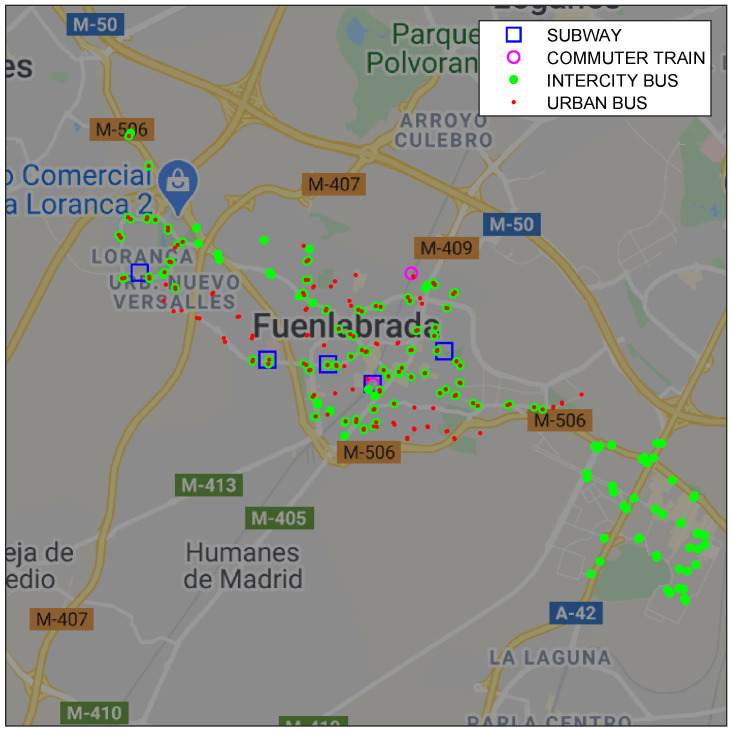
Public transport network in Fuenlabrada. Location of the different stops.

The public transport network in Fuenlabrada integrates 4 different modes: Subway (5 stops), Commuter Train (2), Urban Buses (275) and Intercity Bus to the capital city and other municipalities (414), which include 696 stops in total (Figure 2).

Public transport data were provided by the *Consorcio Regional de Transportes de Madrid* (CRTM), the public agency that coordinates and manages the whole regional public transport network in the Community of Madrid. These data were collected from the smart card system installed globally in the Community of Madrid’s public transport network. The resulting SCD are produced by contactless smart cards that can integrate up to 3 main different titles of transport (pass, multi-ticket, single ticket, etc.). Each validation includes: card ID (anonymized), time stamp, pay-point (a code including the operator, the line, and the stop to represent the boarding station), the type of title (pass, multi-ticket, single ticket, etc. and its modality: tourist ticket, young ticket, senior ticket, children’s ticket, etc.), and the type of discount (large families, disabled persons, etc.). Data were properly pre-processed using Matlab Software to remove invalid entries: duplicates, records with mistakes in some fields, and transactions of people who are not typical public transport users (e.g., subway operators, security people, test tickets, etc.). After pre-processing, we obtained a dataset with 5,825,614 validations from 252,156 different smart cards. Finally, let us indicate that, in order to reduce the driver’s exposure to the virus, there was no obligation to validate on intercity and urban buses during most of the state-of-alarm period (from 23 March to 29 June).

### 3.2. Bluetooth Traffic Monitoring Network and Dataset

**Figure 3:**
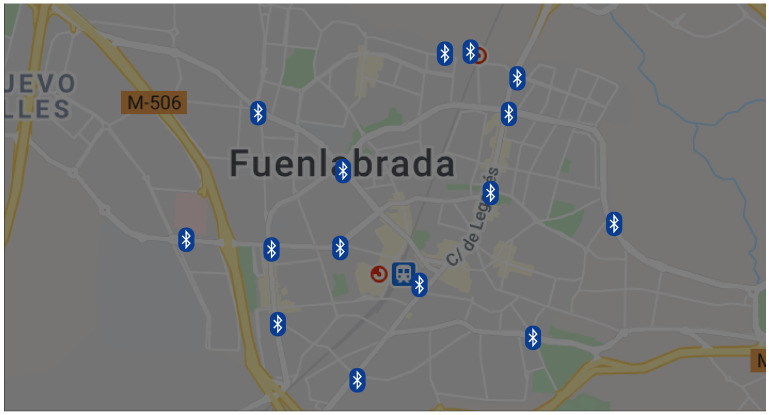
Bluetooth Traffic Monitoring Network in Fuenlabrada. Location of the sensors.

In order to retrieve data regarding private road transport, we deployed a BTMS in Fuenlabrada. These data allowed us to assess average traffic volume changes during the COVID-19 pandemic in the municipality. A BTMS captures information utilizing the Media Access Control (MAC) address of the Bluetooth devices that traverse their coverage area (approximately, a 100-meter radius circular zone). The BTMS is capable of particularly detecting the MAC addresses of hands-free Bluetooth devices in private vehicles, thus providing a precise vehicle identification. As a result, a BTMS captures a statistically representative sample of the total volume of vehicles, which can be used for the reliable estimation of travel times and origin–destination matrices [41]. The aggregation of this stretch-based information provides an overall representation of the traffic in the city. Despite the fact that BTMSs cannot provide exact values for the actual volume of traffic, they can accurately monitor its temporal evolution, thus producing reliable information about the relative changes in the private vehicle’s mobility within the city during the pandemic. For the current study, we use traffic data provided by a BTMS consisting of 15 Bluetooth vehicle identifiers (nodes) that covered the central area of Fuenlabrada as shown in Figure 3.

Each time a Bluetooth node identifies a hands-free Bluetooth device on a vehicle, it generates a *detection*, consisting of the ID of the Bluetooth node, the MAC address of the vehicle, and the time stamp. Raw Bluetooth detections were processed following the algorithm implemented in Python and exposed in [41]. This algorithm first filters out detections that do not correspond to vehicles, using the Dedicated Inquiry Access code (DIAC), thus guaranteeing the specificity of the dataset regarding the private vehicle’s mobility. Next, the methodology integrates multiple detections, produced when a node in a BTMS detects a determined MAC address more than once in a short period of time (for example, a vehicle in a congested scenario). Finally, it removes outliers, i.e., detections that produce travel times that clearly fall outside the common behavior within their particular time slot. This process resulted in a final dataset composed of 2,965,799 vehicle detections from 252,156 different vehicles for all nodes in the network.

## 4. Mobility Changes Produced by COVID-19

COVID-19 has produced evident changes on mobility that have varied throughout the different phases of the pandemic depending on the specific responses that each government put in place in order to control this major health crisis. This section presents a detailed quantitative analysis of this impact on mobility and its evolution, particularized to both private and public transport.

### 4.1. Evolution of Private and Public Mobility

In order to analyze the overall impact of COVID-19 on mobility and perform fair comparisons between transport modes, we fixed a common reference and calculated the corresponding relative changes during each phase of the pandemic with respect to this baseline level. We chose as reference the period of time from 10 February 2020 to 8 March 2020, immediately preceding the explosion of the pandemic in Spain. Relative changes in mobility are calculated dividing the demand in a specific phase by the corresponding demand to this reference period of time. Furthermore, relative changes are evaluated on a weekly basis in order to avoid weekend effects.

**Figure 4:**
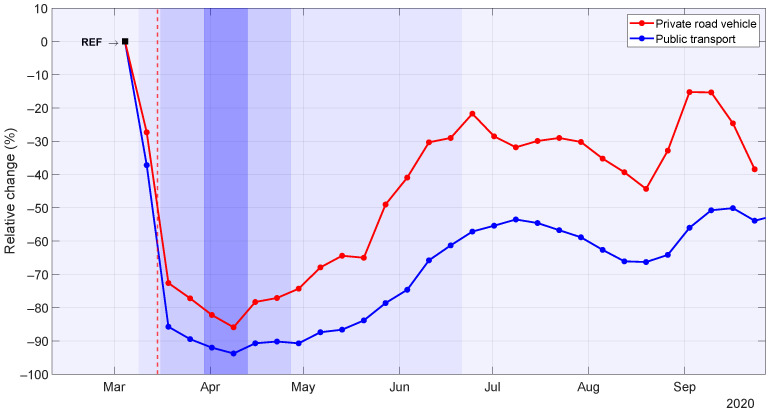
Weekly relative change for private vehicle (red) and public transport (blue) mobility in Fuenlabrada.

Figure 4 shows the relative change in private (red) and public transport (blue) mobility, with respect to the reference. The vertical dashed red line marks the declaration of state of alarm in Spain and the beginning of the national lockdown. In addition, shaded vertical bars represent the different phases of COVID-19 pandemic in Spain shown in Timeline 1 in Figure 1, where the darkest blue corresponds to the halting of all non-essential activity. In general terms, mobility drastically dropped immediately preceding the declaration of the state of alarm, it continued decreasing during the period of economic “hibernation” (with the lowest number of validations), and it finally began to rebound very slowly. We can also observe the seasonal effect of the holiday period during the months of July and August and how mobility started to gradually recover in September, showing values still significantly below those registered before the outbreak of the pandemic.

Analyzing public transport and private mobility separately, we can observe how the latter (red line) dropped to 27% during the week prior to the state of alarm declaration; then, it continued decreasing to almost 72% in the first week of the state of alarm (first darker blue bar); and finally, reached its minimum, 86%, in the the second week of the economic “hibernation” period (darkest blue bar). As for public transport (blue line), results reveal a 37% decrease in the mean daily number of validations during the week prior to the state-of-alarm declaration. Then, it dramatically dropped to almost 86% during the first week of the state of alarm, reaching its minimum (a 95% drop) during the second week of the halting of all non-essential activity period. Comparing public and private mobility, we can observe a lower decrease of the latter, which suggests a possible migration of travelers from public to private transport modes, in line with previous scientific studies that highlighted the perception of public transportation as a potential risk. Whether this pattern is provisional or will consolidate as a definitive change is an important question to be answered in the near future.

Once the deescalation stage started, the public transport demand began to slowly recover. However, it only reached half its pre-pandemic level in September, with a slight drop at the end of the month, when the government imposed new restrictions to control the second wave of the pandemic. On the other hand, private mobility followed a similar overall evolution, but showing a steeper recovery with a drop of only 15% with respect to the reference, during the two first weeks in September of the “new normality” phase.

**Figure 5:**
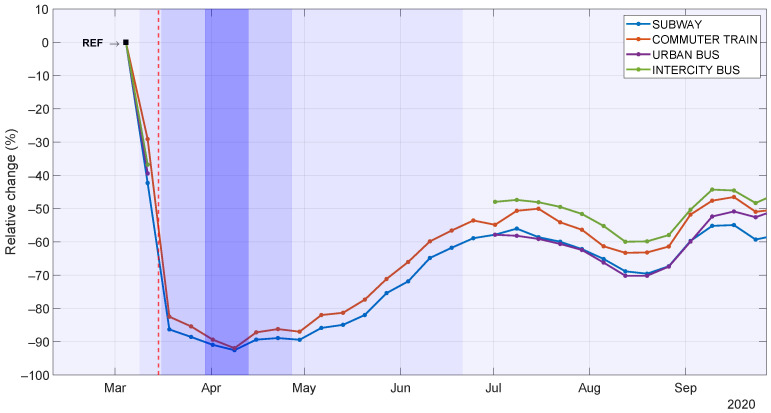
Weekly relative change of daily number of validations per transport mode in Fuenlabrada.

We next proceed to particularize the analysis of the evolution of mobility during the pandemic to each public transport mode. In this study, we do not consider intercity and urban buses from 23 March to 29 June given that there was no obligation for users to validate their smart cards in this period in order to reduce the risk of infection. Figure 5 illustrates the weekly relative change in the daily number of validations per transport mode relative to the baseline level. We observe that all transport modes show a similar decrease, with several relevant peculiarities. First, the commuter train (red) exhibits the lowest drop during the state-of-alarm stage, which reflects a more pronounced dependency of travelers on this transport mode maybe due to a lack of other long-distance transport alternatives. In fact, we observe a similar behavior in intercity bus (green) during the post-state-of-alarm phase. Once the “new normality” phase started (late September), subway (blue) showed the lowest recovery among public transport modes, indicating that travelers continued perceiving it as a potential risk due to its limited ventilation.

### 4.2. Daily Patterns of Private and Public Transport Mobility

In this section, we investigate the temporal changes within a day with the aim of identifying the distinct mobility patterns shown in each phase of the COVID-19 pandemic as illustrated in Timeline 2 in Figure 1. To this end, we present a specific analysis for private mobility and public transport taken as a whole and particularized to each mode.

#### 4.2.1. Private Mobility Daily Patterns

**Figure 6:**
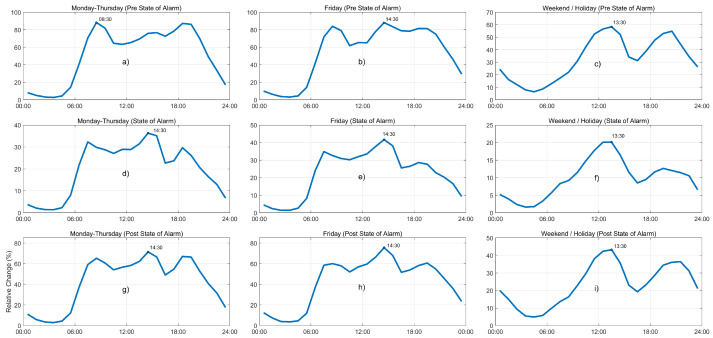
Daily patterns of private vehicle mobility in Fuenlabrada (60-min detections). (**a**) Mondays-Thursdays in pre-state-of-alarm phase. (**b**) Fridays in pre-state-of-alarm phase. (**c**) Weekend/Holidays in pre-state-of-alarm phase. (**d**) Mondays-Thursdays in state-of-alarm phase. (**e**) Fridays in state-of-alarm phase. (**f**) Weekend/Holidays in state-of-alarm phase. (**g**) Mondays-Thursdays in post-state-of-alarm phase. (**h**) Fridays in post-state-of-alarm phase. (**i**) Weekend/Holidays in post-state-of-alarm phase.

Figure 6 shows the daily patterns of private mobility in Fuenlabrada during the three main stages of the pandemic. Information is organized using rows for the three phases in Timeline 2 and columns for different days: Mondays, Tuesdays, Wednesdays, and Thursdays (left); Fridays (center); and weekends and public holidays (right). Additionally, the peak hour showing the highest volume of traffic within the day is highlighted on the curve. Given that a BTMS produces relative measurements of traffic volumes, values express the ratio of 60-min detections over the reference period of time.

These patterns show that the daily distribution of traffic during weekdays (left column) has clearly changed due to the COVID-19 pandemic. During the state-of-alarm scenario, shown in Figure 6d, the morning peak is clearly reduced and it appears an hour before its usual time. The second peak becomes the curve’s new maximum (from 14:00 to 15:00) and the afternoon peak still exists but it is significantly reduced: notice that this third peak in the pre-COVID-19 situation, Figure 6a, was almost as high as the maximum. On the other hand, the shape of the daily pattern for the post-state-of-alarm stage in Figure 6g is similar to that of the previous phase, Figure 6d. It seems that the afternoon peak gains importance, but the most relevant peak in the day is the second one again. This pattern is different from the one in the pre-state-of-alarm phase Figure 6a, which shows an evident change in mobility patterns that cannot be observed by just comparing the overall figures as in Figure 4.

We extract similar conclusions from results corresponding to Fridays (central column). During the state-of-alarm phase, Figure 6e, the morning peak is much lower and appears one hour sooner, and still does not recover its value and original position during the “new normality” phase, Figure 6h. The afternoon peak almost disappears in the second scenario, which reflects that this maximum is related with leisure activities and recreational trips that were canceled during the state-of-alarm situation. Finally, we observe that the shape of weekend and holiday daily public transport patterns in pre- and post-pandemic scenarios, Figure 6c,i, respectively, are rather comparable, with their maximums at 13:00. On the other hand, during the hardest times of the lockdown period, Figure 6f, we can see that the afternoon peak significantly reduces its value because of the closure of recreational activities. The effect of the hard lockdown obviously modified the mobility purposes, which reduced to just commuting.

The results show that the distribution of private vehicle mobility over the course of a day was significantly modified by the lockdown and preventive measures in the specific case of Fuenlabrada. This finding shows a distinct behavior relative to other studies such as [27], where the authors assess the impact of the preventive measures on traffic mobility in Qatar. They observed an overall 30% reduction referred to their baseline, but which did not alter the hourly distribution of traffic. On the other hand, the results in [3] show a softer decay in the morning peak compared to that in the afternoon; in our case, morning and afternoon peaks exhibit the most pronounced reductions, falling below the mid-day peak. These distinct findings confirm the specificity of the behavioral changes that the particular preventive measures produced in different cities around the world.

#### 4.2.2. Public Transport Mobility Daily Patterns

**Figure 7:**
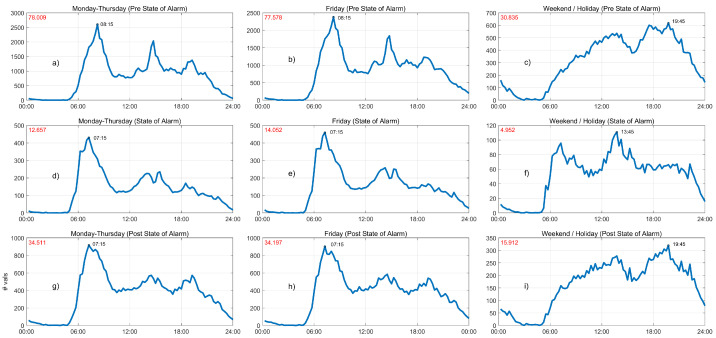
Daily patterns of public transport in Fuenlabrada (15-min validations). (**a**) Mondays-Thursdays in pre-state-of-alarm phase. (**b**) Fridays in pre-state-of-alarm phase. (**c**) Weekend/Holidays in pre-state-of-alarm phase. (**d**) Mondays-Thursdays in state-of-alarm phase. (**e**) Fridays in state-of-alarm phase. (**f**) Weekend/Holidays in state-of-alarm phase. (**g**) Mondays-Thursdays in post-state-of-alarm phase. (**h**) Fridays in post-state-of-alarm phase. (**i**) Weekend/Holidays in post-state-of-alarm phase.

Figure 7 illustrates the average daily ridership patterns calculated from validations aggregated every 15 min. The organization of the figure keeps rows for the phases of the pandemic and columns for the types of day. The number in red at the top left corner indicates the average of number of daily validations in the specific period of time; in addition, the time when validations reached their maximum is shown on the curve. The graphs in Figure 7 use specific scales to allow the direct comparison of the shapes of daily patterns within each phase of the pandemic.

Observing the left column (Monday to Thursday) we can derive two relevant findings. First, we can confirm the drastic drop in the mean daily number of validations since the declaration of the state of alarm, from roughly 78,000 (top) to 12,600 (center), which only recovered to slightly more than 34,000 (bottom) during the “new normality” phase. Second, we notice that the shape of the patterns is also different. During the state-of-alarm scenario shown in Figure 7d, the morning peak (related to commuting) appears an hour before its usual time (7:15). In addition, the mid-day peak still exists but now it shows two distinct local maxima. The third peak in afternoon almost disappears. On the other hand, the shape of the mobility pattern for the post-state-of-alarm stage in Figure 7g is quite similar to that of the previous phase. It seems that the afternoon peak reappears, but it is evident that this pattern is different from the one in the pre-pandemic situation.

We can extract similar conclusions for the average daily pattern on Fridays (central column). Fridays show almost identical patterns to those found in Monday to Thursday, which is an exception to the typical behavior observed for other municipalities and neighborhoods in the Community of Madrid, where Friday is different from other weekdays. During the state-of-alarm phase in Figure 7e, the morning peak appears also an hour before it did during the pre-state of alarm, and still does not recover its original position during the “new normality” phase in Figure 7h. Finally, we observe that the shape of weekend and holiday daily public transport patterns in pre- and post-COVID-19, Figure 7c,i, respectively, are quite similar, with their maximum at 19:45. On the contrary, these models are substantially different from the one corresponding to the state-of-alarm stage in Figure 7f, in which we can observe morning and mid-day peaks that resemble a typical working day behavior. This makes sense since essential workers were the only travelers forced to use public transport on weekends and holidays, in this period of time.

**Figure 8:**
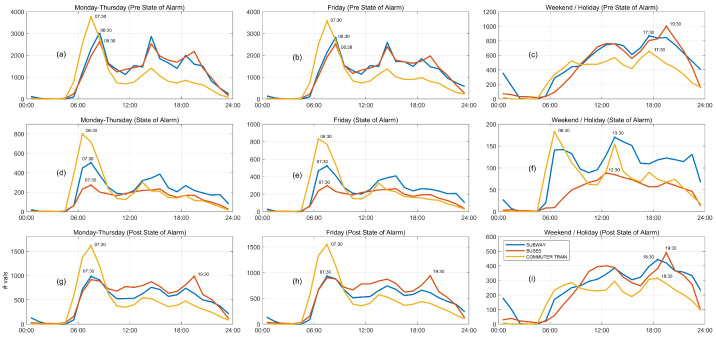
Daily patterns of the main public transport modes in Fuenlabrada (60-min validations). (**a**) Mondays-Thursdays in pre-state-of-alarm phase. (**b**) Fridays in pre-state-of-alarm phase. (**c**) Weekend/Holidays in pre-state-of-alarm phase. (**d**) Mondays-Thursdays in state-of-alarm phase. (**e**) Fridays in state-of-alarm phase. (**f**) Weekend/Holidays in state-of-alarm phase. (**g**) Mondays-Thursdays in post-state-of-alarm phase. (**h**) Fridays in post-state-of-alarm phase. (**i**) Weekend/Holidays in post-state-of-alarm phase.

As complementary information to that presented in Figure 7, we show the average daily ridership patterns for the three main transport modes in Fuenlabrada in Figure 8: subway (blue), urban and intercity buses (red), and commuter train (yellow). Let us focus our analysis on weekdays. First, in Figure 8d, we can observe that the subway’s morning peak appears an hour before its usual time in the pre-state-of-alarm period, while the mid-day peaks reduces its level. During the post-state-of-alarm phase (blue curve in Figure 8g) the subway morning peak does not return to its usual time yet. Second, we notice that the bus pattern substantially changes during the state-of-alarm phase (red curve in Figure 8d), indicating a relatively flat demand until the afternoon, a pattern shape that in general extends to the “new normality” phase (Figure 8g). Third, the commuter train patterns experience the least relevant changes in shape during workdays (yellow curve in left and central columns in Figure 8) during the course of the pandemic. This could be due to the fact that long-distance travelers, who typically use the commuter train, have fewer alternatives, thus being forced to continue using public transport even in an extreme situation such as a pandemic. This commuter train morning peak returns to its usual time (07:30) during post-state-of-alarm phase and gains importance although it does not reach its pre-pandemic levels. Furthermore, the commuter train pattern in weekends and holidays during the state-of-alarm phase (yellow in Figure 8e) replicates the one we found in weekdays, as traveling was limited to essential workers.

## 5. Prediction of Public Transport Demand in COVID-19 Times

Public transport is perceived as a potential risk of infection. This is reflected on actual data by the steeper drop and the slower recovery of its use compared to private mobility. Public transport implies sharing a frequently crowded and reduced space (buses, coaches, and stations) with limited ventilation. Consequently, in order to avoid crowds and respect the safety distance, we need to optimize the public transport services. This optimization often requires the estimation of future states so that the service can be adapted to the expected demand. As a contribution to this objective, we have developed a predictor of the future demand of public transport, based on the daily patterns we have extracted.

### 5.1. Methodology

To this end, we have created specific predictors for the three main modes of transport: subway, commuter train and bus (both urban and intercity). Given the distinct mobility patterns shown in each period of the pandemic (Timeline 2 in Figure 1), we have created specific predictors for each phase that adapt to each particular behavior. The output variable of each of these predictors was exactly the future number of validations to be registered in a specific hour per mode. Models were with a set of six input features: time, day of the week (e.g., Monday), type of day (workday or holiday), and number of validations in the previous hour on each transport mode (subway, commuter train and bus).

**Figure 9:**
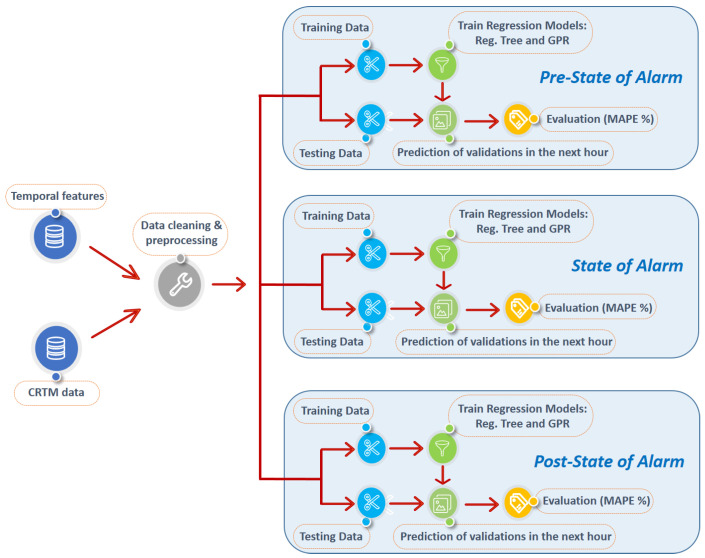
Flowchart of public transport demand prediction algorithm; one for each temporal scenario.

For each period of the pandemic, we used the same amount of data (one month) in order to perform fair comparisons among them; specifically February for the pre-state of alarm, July for the post-state of alarm, and 13 April to 17 May for the state of alarm (this last period was chosen to avoid extreme situations such as the economic hibernation). We pre-processed these data removing errors in the registration of the validations and nighttime periods extending from 22:00 to 06:00, in which some transport modes close or show a negligible demand. We trained our data-driven predictors with data corresponding to the first 21 days in each period of time, using the rest for testing. Figure 9 illustrates a scheme of the predictors (one for each scenario) of future validations we implemented with the training and testing phases characteristic of a machine learning problem.

The prediction of future public transport demand sets out a regression problem, whose output is a numerical signal (number of validations). Thus, we tested different regression approaches observing that *Regression Trees* [42] and *Gaussian Process Regressor* (GPR) [43] show the best performance. The former is one of the most popular algorithms due to its simplicity. A general regression tree is generated when each decision node in the tree contains a test on some input variable’s value. The terminal nodes of the tree contain the predicted output values of the variable. This model is trained through a process known as binary recursive partitioning, which follows an iterative method that splits the data into partitions or branches, and then continues splitting each partition into smaller groups as it moves up each branch. Since the tree can significantly grow from the training set, this approach typically suffers from overfitting. We applied tree pruning in order to reduce the risk of overfitting by verifying the predictive utility of all nodes of a regression tree. On the other hand, GPR is a more complex, non-parametric and probabilistic approach, which is gaining popularity due to its good results in dealing with small datasets and providing uncertainty estimations together with prediction values. A Gaussian process is a probability distribution over possible functions that fit a set of points. The prior knowledge of the form of these functions can be integrated by using kernel functions. In our case, we used an exponential kernel with feature normalization of variables. Finally, we utilized the Mean Absolute Percentage Error (MAPE -%-) as the validation metric. Prediction models were successfully trained and validated using Matlab’s *Statistics and Machine Learning Toolbox*, on an eight-core processor Intel i7-7700HQ (2.8 GHz) with 16 GB of RAM memory.

### 5.2. Performance Evaluation

**Figure 10:**
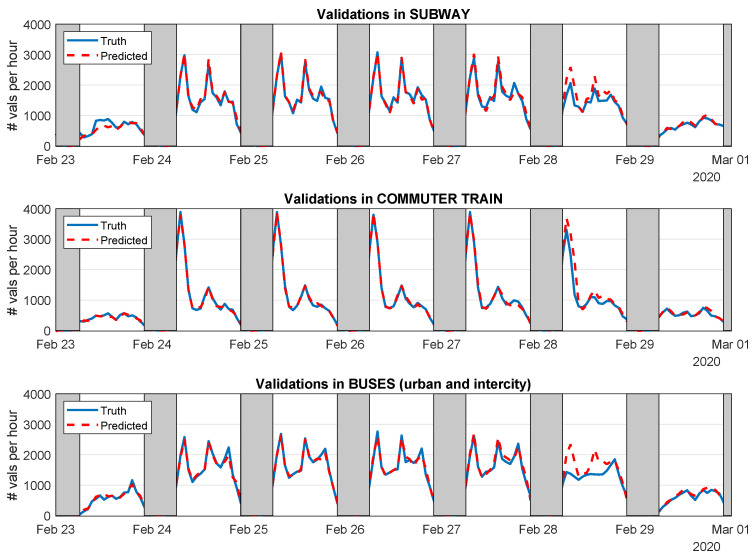
True and predicted demand of each transport mode applying the GPR Model to the pre-state-of-alarm scenario.

In order to illustrate the overall performance of the predictors we have developed, Figure 10 shows the predicted values generated by the GPR model (red in dash line) compared with the actual demand (blue), in the pre-COVID-19 scenario, for each of the three transport modes: subway (top), commuter train (center) and bus (bottom). Gray shaded bars represent nighttimes. We can see how the constructed predictors are capable of correctly forecasting the daily mobility patterns. Among them, Friday 28 February 2020 shows the lowest performance given that it had the peculiarity of being a working day but not a school day, which obviously affects the usual mobility patterns.

**Figure 11:**
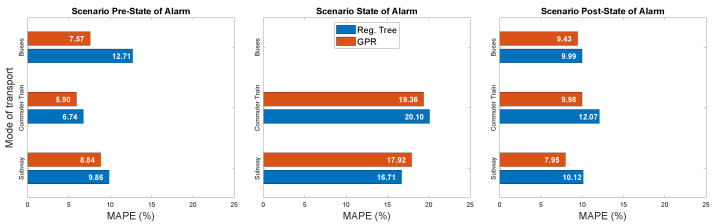
Prediction results (MAPE) for Regression Trees and GPR.

This first qualitative validation of the predictors is extended to a quantitative performance evaluation in Figure 11. Results show the MAPE achieved by Regression Trees (blue bars) and GPR (red bars) applied to the three main transport modes (displayed vertically) during each phase of the pandemic: pre-state of alarm (left), state of alarm (center) and post-state of alarm (right). We do not include predictions for buses during the state of alarm given that there was no obligation to validate on them in this period.

We can observe that GPR achieves values of MAPE below 10% for every transport mode in the pre- and post-state-of-alarm scenarios, reaching 5.9% for commuter trains during the pre-state of alarm. Regression trees also show good performance with MAPEs always below 12% for these two scenarios. Public transport demand in pre- and post-pandemic scenarios exhibited stable patterns, which allowed GPR’s probabilistic nature to outperform other parametric models such as regression trees. The application of these predictors to the state-of-alarm scenario produced performance results with higher MAPE, which are still below 20% in the case of GPR. This is due to the greater variability on the public transport use during this period of time, with different inner phases characterized by distinct restrictions imposed as preventive measures.

### 5.3. Distinct Mobility Patterns in Each Phase of the Pandemic

As the presented results show, the predictors we developed are capable of extracting the inherent behavior of each transport mode during each specific phase of the pandemic. They learn from the underlying patterns in the data and construct accurate forecasts of the future demand. Thus, we can highlight the distinct mobility patterns found on every phase of the pandemic by taking a predictor that was trained with the dataset specific to one scenario and applying it to a different one.

**Figure 12:**
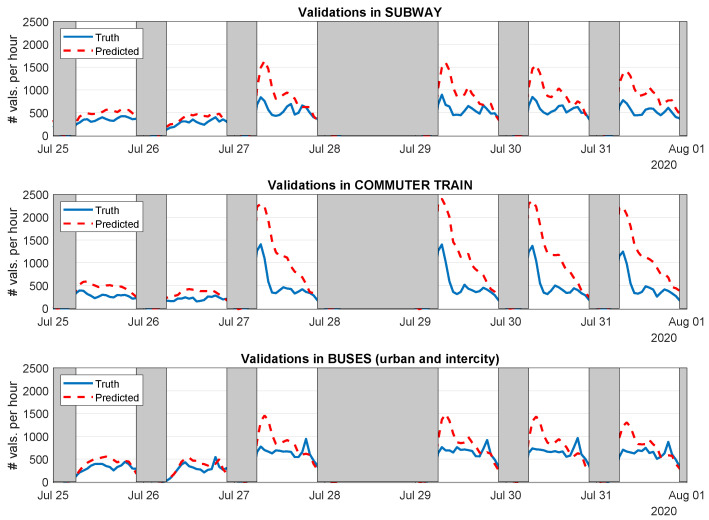
True and predicted demand of each transport mode using the GPR Model trained with data from the pre-state-of-alarm scenario and applied to the post-state-of-alarm period.

Let us first show the qualitative results of this process. Figure 12 displays the predictions performed by the GPR model trained with data from the pre-state-of-alarm period, applied to the post-state-of-alarm phase (we removed from the experiments 28 July as it showed repeated problems in the validation data). In this case, the model was unable to predict neither the expected number of validations, nor even the correct shape of the public mobility pattern.

**Figure 13:**
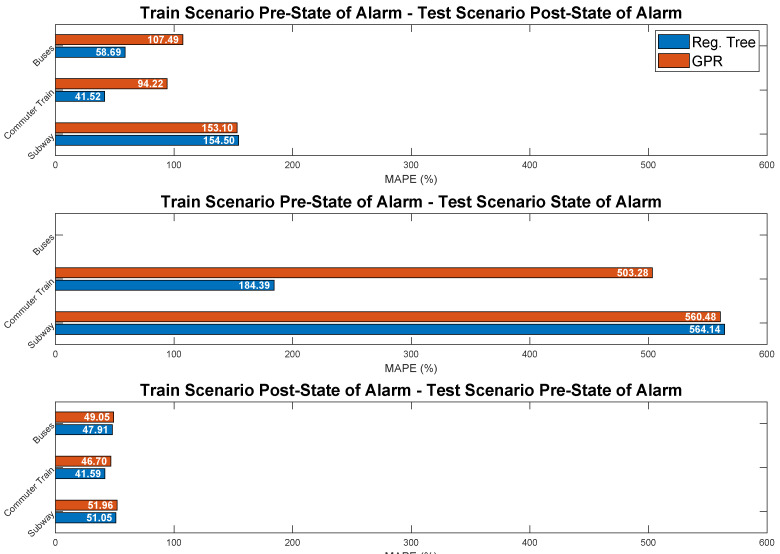
Prediction results (MAPE) for Regression Trees and GPR trained and tested with data belonging to different scenarios.

In addition, this effect is also shown in qualitative terms (MAPE) in Figure 13. The title of each subplot specifies the scenarios we used for training and testing in each case. As expected, the performance of the models drastically drops due to the extremely different use of public transport that characterized each scenario. This led to MAPE values close to 50% at best (post-state-of-alarm model applied to pre-state-of-alarm scenario), drastically increasing above 560% in other combinations. In view of these results, it is evident that the restrictions imposed during each phase of the pandemic and the subsequent behavior of users in regards to public transport severely affected the mobility patterns, confirming the results presented in Section 4.2.2 and other related works in the field.

## 6. Conclusions, Limitations, and Future Research

COVID-19 is changing the travel behavior and disrupting the public transport demand throughout the world. This data-driven study quantifies this impact of the pandemic in the specific case of the internal mobility in a mid-size city in Spain, Fuenlabrada. Significant changes in private mobility and public transport are observed in COVID-19 times, and the implications of these variations on short-term prediction of public transport demand are also investigated in our work.

Our analysis adopted a data-driven approach, using eight months of real data covering all relevant phases of the pandemic in Spain. Results show a drastic fall in the mobility, reaching the maximum decrease in the second week of the halting of all non-essential activity period. In this phase, public and private mobility suffered decreases of 95% and 86%, respectively. The latter recovered faster than the former and after the first months of the “new normality”, it reached similar values to those registered before the outbreak of the pandemic (15% reduction, whilst public transport could only reach 50% of its usual demand). Furthermore, our findings suggest that travelers may be switching from public to private transport modes, which could be due to the perception of the former as a potential risk of infection.

Our results show similar performance to those reflected by other studies such as [3,4] in the cities of Santander (Spain) and Budapest (Hungary), respectively. This fact supports the validity of the models we obtained. We observe an almost identical behavior as in Santander, where the analysis revealed an overall mobility fall, being less important in the case of the private car. Public transport users in Santander dropped by up to 93% (95% in our case) while private vehicle mobility fell in some periods by up to 85% (86% in Fuenlabrada). However, if we compare our results with a bigger city such as Budapest, we clearly observe more important differences. Results for the case of Budapest show that public transport experienced again the greatest reduction in demand but only reaching 80%, whilst car usage shows also a maximum drop of 61%. Consequently, it seems that the COVID-19 impact on the mobility in bigger cities is not as relevant as in mid-size cities such as Fuenlabrada or Santander.

In addition, we performed a detailed analysis of the impact of the pandemic on each individual transport mode and conclude that long-distance transport modes, such as commuter trains, exhibit the least important decays during the state-of-alarm stage, which may reflect the lack of other alternatives to cover these trips. We also study the hourly distribution of public transport and private vehicle mobility, showing distinct daily patterns among the different phases of the pandemic. In both public and private mobility, the morning peak related to commuting is notably reduced and it appears an hour before its usual time, while the level of the third peak in the afternoon is significantly lower (almost disappearing). Finally, the shape of the daily mobility patterns in the post-state-of-alarm stage is quite similar to that of the previous phase.

In the light of these results, we developed predictors of public transport demand, adapted to the specific behaviors of each phase of the pandemic. The GPR predictor achieved a high prediction performance with MAPE values below 10% for pre- and post-state-of-alarm phases and below 20% for the state-of-alarm scenario, where mobility patters are more variable. This accurate short-term predictors could significantly contribute to design transport policies capable of adapting to crisis and improve the quality of service of transportation systems in normal situations. Despite its contribution to the characterization of COVID-19’s impact on mobility in mid-size cities and the prediction of future demand, our work shows a spatial and temporal limitation inherent to its intrinsic data-driven nature. In this regard, the datasets we have used are specific to a certain municipality and temporal range. Considering the spatial component, further analysis performed on new datasets collected in other cities and towns throughout the world will surely shed light to particular aspects of the effects of COVID-19 on mobility. On the other hand, unfortunately, the pandemic is still an ongoing health issue; thus, the scientific community will have to keep on analyzing the temporal evolution of its impacts. COVID-19 is a global and open-ended crisis, which will imply a work in progress in the research field.

The world in general and mobility in particular are facing an unprecedented transformation in a very short period of time due to COVID-19. To tackle the effects of this pandemic, countries have implemented a range of preventive policies, including stay-at-home lockdowns, school and workplace closures and restrictions on public transport. Our work and other previous research show that these policies have been effective in reducing human mobility but have also contributed to a potential risk regarding its sustainability in urban areas, with an evident migration from public to private transport modes. These effects need to be fully investigated to determine whether cities will recover pre-pandemic mobility demand patterns and how this could be achieved. New alternatives, such as teleworking, videoconferencing or e-commerce have demonstrated the viability of new approaches to mobility that avoid unnecessary trips, respecting the environment and increasing comfort in cities. We are witnessing a time of change that we can leverage to introduce transforming actions to create resilient and sustainable cities, based on technology and knowledge. Our work is in line with this objective and contributes to the state of the art in the field, providing a deep analysis about mobility in a mid-size city to cover the existing gap in the current literature. 

## Data Availability

Not applicable.
